# Characterization of Transcription Factor Networks Involved in Umbilical Cord Blood CD34^**+**^ Stem Cells-Derived Erythropoiesis

**DOI:** 10.1371/journal.pone.0107133

**Published:** 2014-09-11

**Authors:** Biaoru Li, Lianghao Ding, Chinrang Yang, Baolin Kang, Li Liu, Michael D. Story, Betty S. Pace

**Affiliations:** 1 Department of Pediatrics, Hematology/Oncology Division, Georgia Regents University, Augusta, Georgia, United States of America; 2 Department of Radiation Oncology and Simmons Comprehensive Cancer Center, University of Texas Southwestern Medical Center, Dallas, Texas, United States of America; 3 Department of Molecular and Cell Biology, University of Texas at Dallas, Richardson, Texas, United States of America; French Blood Institute, France

## Abstract

Fetal stem cells isolated from umbilical cord blood (UCB) possess a great capacity for proliferation and differentiation and serve as a valuable model system to study gene regulation. Expanded knowledge of the molecular control of hemoglobin synthesis will provide a basis for rational design of therapies for β-hemoglobinopathies. Transcriptome data are available for erythroid progenitors derived from adult stem cells, however studies to define molecular mechanisms controlling globin gene regulation during fetal erythropoiesis are limited. Here, we utilize UCB-CD34^+^ stem cells induced to undergo erythroid differentiation to characterize the transcriptome and transcription factor networks (TFNs) associated with the γ/β-globin switch during fetal erythropoiesis. UCB-CD34^+^ stem cells grown in the one-phase liquid culture system displayed a higher proliferative capacity than adult CD34^+^ stem cells. The γ/β-globin switch was observed after day 42 during fetal erythropoiesis in contrast to adult progenitors where the switch occurred around day 21. To gain insights into transcription factors involved in globin gene regulation, microarray analysis was performed on RNA isolated from UCB-CD34^+^ cell-derived erythroid progenitors harvested on day 21, 42, 49 and 56 using the HumanHT-12 Expression BeadChip. After data normalization, Gene Set Enrichment Analysis identified transcription factors (TFs) with significant changes in expression during the γ/β-globin switch. Forty-five TFs were silenced by day 56 (Profile-1) and 30 TFs were activated by day 56 (Profile-2). Both GSEA datasets were analyzed using the MIMI Cytoscape platform, which discovered TFNs centered on KLF4 and GATA2 (Profile-1) and KLF1 and GATA1 for Profile-2 genes. Subsequent shRNA studies in KU812 leukemia cells and human erythroid progenitors generated from UCB-CD34^+^ cells supported a negative role of MAFB in γ-globin regulation. The characteristics of erythroblasts derived from UCB-CD34^+^ stem cells including prolonged γ-globin expression combined with unique TFNs support novel mechanisms controlling the γ/β-globin switch during UCB-derived erythropoiesis.

## Introduction

UCB-CD34^+^ stem cells (UCB-SC) represent a powerful paradigm for exploring many aspects of cell biology and hold considerable promise as a therapeutic option for hematopoietic stem cell transplantation and *ex vivo* gene therapy. A large body of evidence suggests that UCB-SC have unique biological characteristics including growth kinetics, morphology, phenotype, differentiation potential and engraftment capacity when compared to adult bone marrow-derived CD34^+^ stem cells [1]–[2]. These features provide the impetus for developing UCB-SC for pre- and post-natal therapy for malignant [3] and inherited β-hemoglobinopathies such as thalassemia major [4] and sickle cell disease [5]. However, the molecular mechanisms that control UCB-SC derived erythropoiesis including globin gene regulation remain unclear. Therefore, we utilized UCB-SC induced to undergo erythroid maturation as a model for characterizing the fetal transcriptome to gain insights into globin gene regulation. The major protein produced during erythropoiesis is hemoglobin produced from five functional genes (ε, Aγ, Gγ, δ, and β-globin) located in the β-locus on chromosome 11, expressed in a stage-specific manner during development [6]. The normal switch from γ- to β-globin (γ/β-globin) gene expression occurs after birth by one year of life.

To date, gene profiling during human erythroid differentiation using various liquid culture systems have been published, contributing insights into differentially expressed genes and the molecular control of lineage commitment [7]–[8]. For example, Merryweather-Clarke et al. used peripheral blood mononuclear cells combined with fluorescence-activated cell sorting to generate expression data from erythroid progenitors generated in culture [9]. Our laboratory recently characterized the transcriptome associated with the γ/β-globin gene switch in erythroid progenitors derived from adult bone marrow CD34^+^ stem cells [10]. Although an increasing number of studies have addressed the transcriptome of adult erythropoiesis, limited data are available for erythropoiesis in fetal progenitors and the TFNs involved in the γ/β-globin switch.

The impetus for defining TFNs involved in hemoglobin switching is to develop strategies for fetal hemoglobin (HbF; α2γ2) induction to treat sickle cell anemia caused by an A to T mutation in the γ-globin chain. This mutation leads to hemoglobin S production which is subjected to non-covalent polymerization under low oxygen conditions. Many studies describing naturally occurring mutations producing hereditary persistence of HbF expression, document the amelioration of clinical symptoms in sickle cell disease [11]. Although hydroxyurea therapy has been used to successfully induced HbF in the majority of sickle cell patients [12], [13], defining global mechanisms of γ-globin regulation have the potential to provide alternative approaches for HbF induction in non-responsive individuals.

The majority of studies to identify regulators of γ-globin transcription have utilized cell lines [14], adult stem cells [15] or animal models [16]. Recent human genetic studies revealed an association of mutations in the BCL11A gene and inherited HbF levels [17] which were demonstrated to be involved in the γ/β-globin switch in transgenic mice [18]. Subsequent studies demonstrated that KLF1, an erythroid-specific protein which directly activates β-globin [19] also regulates BCL11A [20], [21]. The latter is a repressor that binds to sequences in the locus control region (LCR) and downstream of Aγ-globin in adult erythroid progenitors to silence γ-globin expression through protein-protein interactions with Sox6 [22]. In this study, we used UCB-SC as a model to characterize the transcriptome and TFNs involved in globin gene regulation during fetal erythropoiesis. Gene Set Enrichment Analysis identified 45 TFs silenced by day 56 (Profile-1) and 30 TFs activated by day 56 (Profile-2) in culture. Subsequent MIMI Cytoscape platform analysis discovered novel TFNs centered on KLF4 and GATA2 (Profile-1) and KLF1 and GATA1 for Profile-2 genes. Subsequent shRNA studies in human erythroid progenitors generated from UCB-CD34^+^ cells supported a negative role of MAFB in γ-globin regulation.

## Materials and Methods

### One-phase Erythroid Culture

UCB-derived CD34^+^ stem cells (STEMCELL Technologies, Vancouver, Canada) were grown in the one-phase liquid culture system as previously published [23]. Briefly, cells were cultured in αMEM containing 30% fetal bovine serum (Atlanta Biologicals, Atlanta, GA), stem cell factor (50 ng/mL), interleukin-3 (10 ng/mL) and erythropoietin (4 IU/mL) starting on day 0. Three million cells were harvested every 7 days for the different studies. For biomarkers analyses single layer cell smears were made by cytospin preparations and fixed in 4% paraformaldehyde. Cells were stained with FITC conjugated anti-CD34 and anti-CD235a antibodies, and PE conjugated anti-CD71 antibody (eBioscience, San Diego, CA). The number of biomarker positive cells was counted using a florescent microscope (Zeiss, Avix Vision 4.8); at least 500 cells were counted per slide in triplicate for each time point analyzed.

### Reverse transcription-quantitative PCR (RT-qPCR) analysis

The mRNA levels of γ-globin, β-globin, and glyceraldehyde-3-phosphate dehydrogenase (GAPDH) were measured as previously published [24]. Total RNA was extracted from 3×10^6^ cells and the different cDNAs were prepared using the Improm-II RT system (Promega, Madison, WI). The γ-globin, β-globin, and GAPDH mRNA levels were quantified by Sybergreen qPCR (iCycler 95 iQ, Bio-Rad).

### Illumina BeadChip Microarray Analysis

Total RNA isolated on day 21, 42, 49 and 56 was used for microarray analysis on the Illumina HumanHT-12 V4 Expression BeadChip platform (Illumina, Inc., San Diego, CA) as previously published [25]. Quality checked cRNA was hybridized to the Illumina BeadChip using streptavidin-Cy3 for detection and chips were scanned on an Illumina Beadstation. The raw data are available through the National Center for Biotechnology Information Gene Expression Omnibus database, accession number GSE49438.

### Microarray Confirmation

RT-qPCR was used to confirm microarray data as previously published [10]. Gene specific primers were designed using Primer3 software. To validate the microarray data we chose a subset of Profile-1 and Profile-2 genes. The correlation coefficient (R^2^) and confidence intervals were generated using the Student's *t*-test, p<0.05.

### Principal Component Analysis (PCA)

The raw data obtained from the Illumina Beadstation were summarized as probe level signal intensities using Illumina BeadStudio v2.1.3, then background subtraction and quantile normalization were completed using the MBCB (Model-Based Background Correction for BeadArrays) algorithm [26]. After data normalization, we defined gene expression patterns during fetal erythropoiesis and performed time-course analysis with PCA (NIA Array Analysis Tool) as previously published [10], [27]. The data were analyzed at the >1.5-fold change levels in a time-course manner on days 21, 42, 49 and 56 to define two major gene expression profiles.

### Gene Set Enrichment Analysis (GSEA)

We performed GSEA [28] to enrich for transcription factors with >1.5-fold changes in expression between day 21 (high γ-globin) and day 56 (high β-globin) by PCA. GSEA is a computational method that determines whether an *a priori* defined set of genes shows statistically significant, concordant differences between two biological states. The method derives its power by focusing on gene sets that share common biological function, chromosomal location, or regulation. For our GSEA analysis we interrogated three gene sets including TF activity, TF complex and DNA binding. For computing the statistical significance of a biological category, 100 permutations were performed with phenotype comparison ranking established by Signal2Noise, a metric parameter for enrichment and the Meandiv test for normalization models. Two measures were generated by GSEA including the enrichment score (ES) and the gene ranked list metric. The ES reflects the degree to which a gene set is overrepresented at the top or bottom of a ranked list of genes. Our ranked list metric measures a gene's correlation with the Profile-1 or Profile-2 phenotypes. After GSEA the TFs identified were analyzed by hierarchical clustering using the BRB ArrayTool to confirm expression patterns during erythropoiesis.

### TESS and TFSEARCH analysis

To search for putative TF binding motifs in the β-locus on chromosome 11, TESS and TFSEARCH software tools were used. The Genome Browser (http://genome.ucsc.edu/) was employed to confirm motif coordinates in the Human Genome, version Hg 19.

### Cytoscape Michigan Molecular Interaction (MiMI) Analysis

Cytoscape is an open source software platform for visualizing complex networks and integrating them with attribute data. MiMI is a plugin for Cytoscape to study molecular interactions from the MiMI database and to display the interaction network(s) in Cytoscape [29]. This approach was used to display the TFNs established using genes identified by GSEA.

### ENCODE Analysis

Data tracks from the ENCODE project were downloaded and analyzed using the UCSC Genome Browser. Our analysis covered the genomic region from 5,237,658 to 5,318,750 (the β-locus on chromosome 11). The gene transcription (RNA-seq), histone modification, and ChIP-seq (*in vivo* TF binding) tracks were generated from data produced by the ENCODE consortium.

### Lentivirus-mediated shRNA Gene silencing

GIPZ lentiviral shRNA particles were purchased from Thermo Fisher Scientific Inc. (Waltham, MA). KU812 cells (90,000) were transduced with 20 MOI of lentivirus particles in serum-free media for 4 hr and then 10% fetal bovine serum was added. Puromycin (0.6 µg/ml) was added on day 2 and selection performed for 5 days. The cells were harvested for RT-qPCR using gene-specific primers purchased from SuperArray (Qiagen, Valencia, CA); relative gene expression levels were calculated using the 2^−ΔΔCT^ method. After lentiviral transduction fluorescent activated cell sorting (FACS) was performed at 48 hr to determine transfection efficiency. The percentage of green fluorescence protein (GFP) positive cells was used to normalize the qPCR data.

### Transduction of erythroid progenitors

Erythroid cells were generated from UCB-SC in culture as described in the One-phase Erythroid Culture section. On day 49 erythroid progenitors (90,000) were transduced with 20 MOI of the different lentivirus particles in serum-free media for 4 hr then cells were grown in complete medium for the duration of culture. Puromycin (0.6 µg/ml) was added at day 51 and cells were harvested at day 56 for GFP expression by FACS to determine transfection efficiency and RT-qPCR analysis. The γ-globin and β-globin gene expression levels were normalized by %GFP positive cells and expressed as a ratio of γ/γ+β and β/γ+β.

### Fluorescent Activated Cell Sorting (FACS) analysis

After virus transductions 300,000 KU812 cells or erythroid progenitors were washed twice with phosphate buffered saline then fixed in 4% paraformaldehyde and permeated with ice-cold acetone/methanol (4∶1). Cells were incubated with anti-γ-globin-FITC antibody (Santa Cruz Biotechnology, Santa Cruz, CA) in PBT (PBS/01%BSA/0.1% triton X100) solution. The labeled cells were analyzed by FACS on a Bectin Dickerson LSR-II flow cytometer (BD Bioscience). All experimental were performed in triplicate of three to five independent viral transductions.

## Results and Discussion

### Normal erythropoiesis is observed in UCB-stem cells

**Figure 1:**
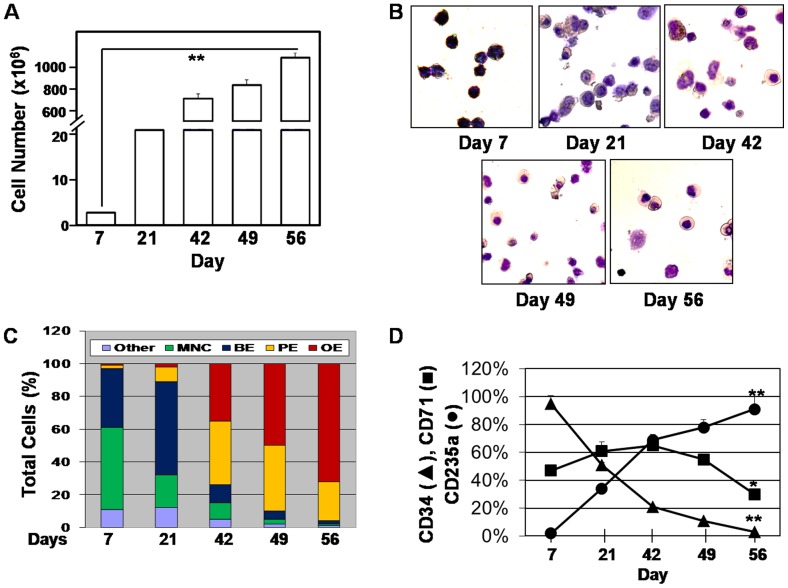
Erythroid progenitor characterization in the one-phase culture system. A) Cells were harvested every 7–14 days and cell counts and viability were performed by trypan blue exclusion. Over 10 billion cells were generated by day 56 in culture. **p<0.01 B) Shown is the cellular morphology demonstrated by Giemsa stain (40X magnification). C) Shown is a summary of the erythroid progenitors counted at the different days after Giemsa stain. At least 500 cells were counted per slide in triplicate. Abbreviation: MNC, mononuclear cells; BE, basophilic erythrpoblast, PE, polychromatophilic erythroblast, OE, orthochromatophilic erythroblast. D) Changes in cell surfaced biomarkers that occurred during fetal erythroid differentiation were measured by cytospin preparation and immunohistochemical stain (See Materials and Methods). *p<0.05.

To study global gene expression patterns in UCB-SC, we used the one-phase liquid culture system as previously published [23]. Cell growth curves, viability, morphology and differentiation biomarkers measured during UCB-SC erythropoiesis displayed a high growth potential by day 56 in cultures (Figure 1A). We observed 72% orthochromatophilic erythroblasts by the end of culture period (Figure 1B and 1C) with greater than 93% viability at the time of cell harvest (Figure S1 in File S1). Loss of CD34 expression and increased CD71 and CD235a expression confirmed erythroid commitment (Figure 1D) and maturation of erythroid progenitors in our system. We did not observe significant enucleation of progenitors by day 56 therefore our system is not optimal for investigating the transcriptome of reticulocytes and mature red blood cells.

### The γ/β-globin switch is recapitulated in UCB-stem cells

**Figure 2:**
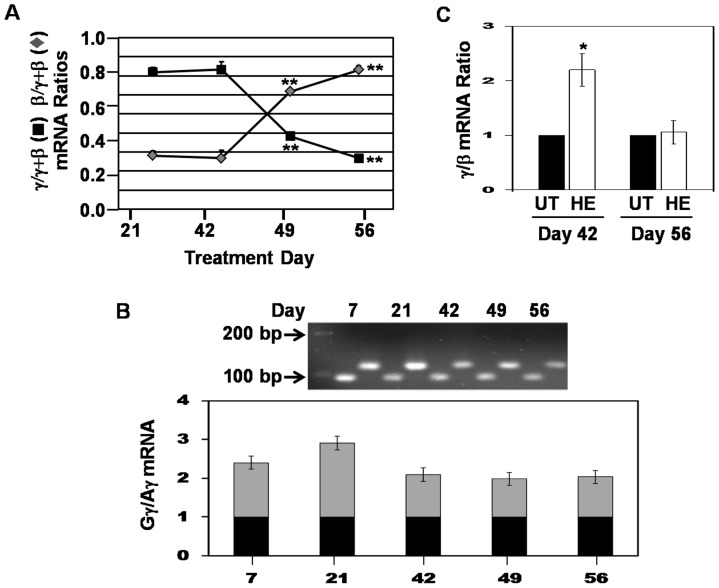
The γ/β-globin switch is recapitulated in UCB-stem cells. A) RT-qPCR analysis was performed at the days shown to determine changes in γ-globin and β-globin mRNA levels after normalization by the internal control GAPDH. B) RT-qPCR was performed with Gγ- and Aγ-globin specific primers to determine the Gγ/Aγ mRNA ratio during erythropoiesis. A representative gel is shown. Gγ-globin expression was normalized to one (black bars) and the relative changes in Aγ-globin mRNA are shown in the gray bars. C) Progenitors were induced on day 42 or day 56 with 50 µM hemin (HE) for 72 hr and then RT-qPCR was completed to measure γ-globin and β-globin mRNA levels.

To substantiate this system as a model to study TFNs involved in hemoglobin switching, γ-globin and β-globin mRNA levels were quantified by RT-qPCR. Before day 14 the γ- and β-globin genes were expressed at low levels (data not shown) however by day 21 when erythroid progenitors reached a significant level (Figure 1C), γ-globin expression predominated with the γ/β-globin switch occurring after day 42 (Figure 2A). This pattern of globin expression in different than adult stem cell erythropoiesis where γ-globin predominates by day 7 and the γ/β-globin switch occurred around day 21 (Figure S2 in File S1) [10]. These data demonstrate the γ-globin gene is active for a longer period in fetal erythroid progenitors suggesting different mechanisms of globin gene regulation might occur in these two cell models.

We observed changes in globin gene expression as progressively more mature erythroblasts appear in culture. However in our system, erythroblasts may be sequentially generated by progenitors generated at different points in culture since CD34^+^ cells are detected up to day 49 (Figure 1D). The latter possibility is supported by the fact that mature red blood cells survive in culture about 7 days but the lack of enucleation in the one-phase culture supports longer survival for erythroblasts. Comparison of the globin genes expressed in cells generated from erythroblasts and CD34^+^ cells purified at different time points is required to clarify this point.

Fetal hemoglobin is a heterogeneous mixture of γ-globin polypeptide chains containing either glycine (Gγ) or alanine (Aγ) at residue 136; Aγ-chains increase from a 3∶1 to 1∶1 Gγ:Aγ ratio during the first year of life. To further substantiate our system we determined the Gγ- and Aγ-globin expression patterns using gene-specific primers. As shown in Figure 2B, the Gγ:Aγ-globin ratio changed from 2.3∶1 at day 7 to 1∶1 by day 42 recapitulating expression patterns observed in the first year of life.

Finally, to investigate γ-globin activation in our system erythroid progenitors were treated with hemin, a known HbF inducer, at two time points corresponding to high γ-globin (day 42) and low γ-globin (day 56) expression. As shown in Figure 2C hemin (50 µM) activated γ-globin 2.2-fold after 72 hr of treatment, which was lost at the later time point. These data support the ability of hemin to further enhance a transcriptionally active γ-globin gene compared to when the gene is silenced at the end of culture. Data supporting the γ/β-globin switch, change in the Gγ:Aγ ratios, and HbF induction by hemin support the capacity of our culture system to define global TFNs associated with hemoglobin switching during fetal erythropoiesis. This type of analysis has not been completed to date. Nevertheless, our system has limitations due to the isolation of RNA from mixtures of erythroblast at the different time points. To address this limitation we performed siRNA functional studies to determine if the TFs identified by bioinformatics analysis have an effect on γ-globin transcription.

### Gene expression profiling during fetal erythropoiesis

To map gene expression profiles we collected samples at day 21, 42, 49, and 56 based on the timing of the γ/β-globin switch (Figure 2A). Using MBCB software, the raw data were normalized for variations in gene expression between replicates (Figure S3 in File S1 and Table S1), and the quality of data at each time point was further studied by regression analysis. The normalized triplicates at day 21 showed R^2^ = 0.99, day 42 R^2^ = 0.96, day 49 R^2^ = 0.98, and at day 56 R^2^ = 0.95 demonstrating good correlation of data reproducibility.

### PCA defines Profile-1 and Profile-2 gene expression patterns

**Figure 3:**
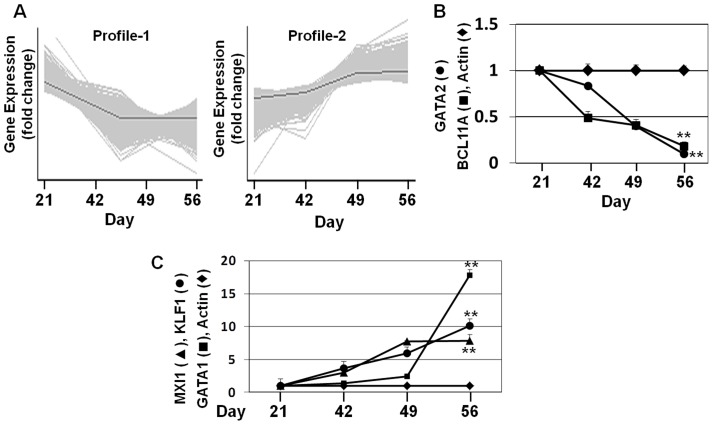
PCA defines major gene profiles during erythropoiesis. A) PCA was performed for gene subsets with >1.5-fold change in expression from day 21 to day 56. The results are shown for the two major gene profiles generated by PCA. The gray line represents the mean value of time course changes in gene expression. B) Microarray data for two known γ-globin regulators were confirmed by RT-qPCR. C) Microarray data for three Profile-2 genes were confirmed by RT-qPCR.

We subsequently generated major gene expression patterns using PCA to build a model to define the TFNs involved in hemoglobin switching. We hypothesized that Profile-1 TFs with an expression pattern similar to γ-globin (genes silenced from day 21 to day 56) might be activators of γ-globin, or conversely repressors of β-globin. Similarly, Profile-2 genes (activated from day 21 to day 56) might be β-globin activators or repressors of γ-globin. Alternatively, TFs with either expression profile may not play a role in globin gene regulation but rather be involved in normal erythroid maturation. The two gene profile subsets were defined using PCA (Figure 3A) and the normalized data were mined by statistical analysis using an F-distribution with ANOVA = 0.01 and a false discovery rate (FDR) = 0.05 (Table S2). We identified 2,568 Profile-1 and 2,458 Profile-2 genes with >1.5-fold change in expression during fetal erythropoiesis. RT-qPCR was completed for a subgroup of TFs known to be involved in globin gene regulation. We observed GATA2 and BCL11A silencing over 56 days (Figure 3B). The findings for BCL11A are opposite of that observed for adult stem cells [30] supporting difference in gene regulation in fetal progenitors. By contrast, gene activation occurred over the culture period for the Profile-2 genes KLF1, GATA-1 and MXI (Figure 3C). We next confirmed the microarray data with RT-qPCR analysis of 25 Profile-1 and Profile-2 genes combined with regression analysis (Table S3). Our results showed good correlation between the microarray and RT-qPCR datasets with R^2^ ranging from 0.72 to 0.79. However, confirmation of Profile-2 genes was better than Profile-1 genes which may reflect prolonged culture period for the latter, a finding reported by other laboratories [31].

### GSEA and TESS/TFSEARCH analysis identify TFs with altered expression during the γ/β-globin switch and predicted binding in the β-locus

**Figure 4:**
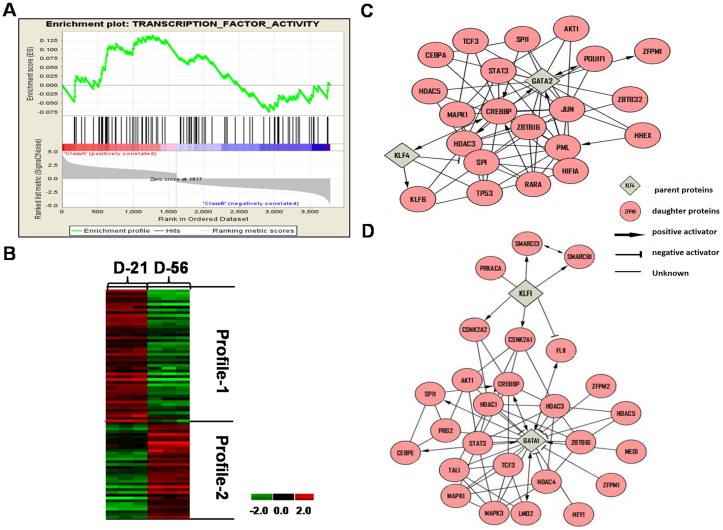
GSEA Analysis. A) Profile-1 (2568) and Profile-2 (2458) genes generated by PCA comparing day 21 to day 56 gene expression levels, were analyzed by GSEA to generate enrichment scores (ES) and a gene rank list. (Top) The top portion of the plot shows the ES for the different TFs as the analysis walks down the ranked gene list. The scores at the peak of the plot (the scores furthest from 0.0) are shown. TFs with a peak at the beginning or end of the ES plot are the most interesting. (Middle) This portion of the figure shows the position of the TFs relative to the ranked list of genes. (Bottom) The bottom portion of the plot shows the value of the ranking metric which measures a TFs' correlation with differential expression on day 21 and day 56. The Class A (Profile-1) genes have positive ES values and Class B (Profile-2) genes correlate with a negative ES value. B) Hierarchical clustering was performed for Profile-1 and Profile-2 genes. Culture day (columns) and genes (rows) were clustered by log-transformed intensity values using correlation distance with similarity metric and centroid linkage. Up-regulated genes are shown in red, repressed genes in green and genes with no change in expression in black. C) Shown is a major TFN generated by Cytoscape analysis of Profile-1 genes. The key is included for interpretation of predicted regulatory interactions. D) Shown is a major TFN generated by Cytoscape analysis of Profile-2 genes. The interaction key is the same as in panel C.

To define TFs involved in the γ/β-globin switch we performed GSEA, which determines whether an *a priori* defined set of genes show concordant differences between day 21 and day 56 of culture. The ES (enrichment score) reflects the degree to which three TFs gene sets are overrepresented at the top or bottom of a ranked list of genes. A positive or negative ES indicates gene enrichment at the top or bottom of the ranked list respectively. We input 2568 Profile-1 and 2458 Profile-2 genes and generated a rank ordered list related to the signal to noise ratio; 3786 genes with >1.5-fold change between day 21 and day 56 were ranked. Shown in Table 1 and Table 2 respectively are 40 positively correlated Class A (Profile-1) and 30 negatively correlated Class B (Profile-2) TFs based on ES generated by GSEA (Figure 4A) supporting possible function in erythropoiesis.

**Table 1:** UCB-stem cell Class A (Profile-1) transcription factors.

Gene No.	GENE SYMBOL	ENRICHMENT SCORE (ES)^1^	GENE RANK^2^
1	WRN	0.13921548	1273
2	RAD51L3	0.13392287	1131
3	HMGB1	0.12708704	1455
4	RAD51C	0.12248376	1420
5	ZHX1	0.12059385	1453
6	MCM2	0.11607464	760
7	ORC1L	0.109770335	660
8	HMGB3	0.10697572	833
9	ORC5L	0.0941495	956
10	WDHD1	0.093303196	996
11	ERCC1	0.09263573	638
12	SP1	0.0846437	1220
13	FANCG	0.084250145	626
14	TCF3	0.07799235	1354
15	AEBP1	0.076171584	1191
16	ATF5	0.076171584	1291
17	RBL1	0.074729405	618
18	UHRF1	0.070319876	1329
19	MYCN	0.06354961	616
20	CCRN4L	0.061169855	1185
21	TFAP4	0.059797958	1128
22	RAD51	0.05145543	574
23	ABI2	0.041926224	565
24	TP53	0.040889304	1068
25	KLF4	0.040889304	1068
26	MXD3	0.03288997	554
27	SCML1	0.030790053	1039
28	GATA2	0.026186744	197
29	FAF1	0.021628574	551
30	ALKBH2	0.021547657	320
31	SREBF1	0.018373493	1018
32	KIF2C	0.016045136	527
33	FOXD1	0.015144873	753
34	EXO1	0.013996358	184
35	ESR2	0.013428025	271
36	TCF7L2	0.012766987	448
37	SCMH1	0.008562001	701
38	ZMYM3	0.006172042	518
39	ZNF367	0.003327756	642
40	ZNF133	0.001190173	1014

1 ES, enrichment score of TFs identified by GSEA using three TF gene sets (TF activity, TF complex and DNA binding). A positive ES indicates the gene expression correlated with a Profile-1 pattern (decreased expression from day 21 to day 56).

2 Gene Rank, gene rank list generated by GSEA using 3786 TFs with >1.5-fold changes in expression from day 21 to day 56 in culture.

**Table 2:** UCB-stem cell Class B (Profile-2) transcription factors.

Gene No.	GENE SYMBOL	ENRICHMENT SCORE^1^	GENE RANK^2^
1	SON	−0.05842792	2994
2	TSC22D3	−0.058295283	2927
3	NFX1	−0.054407794	2935
4	DMC1	−0.053078525	2844
5	ATF3	−0.051295283	2927
6	TRIM22	−0.04250992	3495
7	SOX6	−0.04150992	3495
8	NFKB2	−0.038754087	2935
9	BATF	−0.03710674	3189
10	GATA1	−0.03329886	3749
11	KLF1	−0.032754087	2935
12	FOXC1	−0.030511508	3262
13	SP140	−0.03033415	3212
14	STAT1	−0.02901836	3336
15	MAFB	−0.02863797	3306
16	GLI2	−0.02699749	3520
17	BCL6	−0.02135538	2685
18	ZNF256	−0.0191952	2182
19	SYCP1	−0.017439079	3545
20	CDT1	−0.017186195	2181
21	E2F1	−0.013077641	2999
22	MXI1	−0.012244189	3349
23	IKZF4	−0.008271581	2442
24	HMGA1	−0.007992041	2911
25	CREB1	−0.006540393	2653
26	JUN	−0.005443786	3064
27	OLIG2	−0.003412304	3554
28	SIAH2	−0.002095686	3614
29	HSF1	−0.001728075	2562
30	ZHX2	−0.00001730	2292

1 ES, enrichment score of TFs identified by GSEA using three TF gene sets (TF activity, TF complex and DNA binding). A negative ES indicates genes with a negative correlation to Profile-1 (i.e. Profile-2 gene with increased expression from day 21 to day 56).

2 Gene Rank, gene rank list generated by GSEA using 3786 TFs with >1.5-fold changes in expression from day 21 to day 56 in culture.

Of the Profile-1 genes, we identified GATA2 and KLF4 which are known to be positive regulators of γ-globin supporting the predictive model and published data from our laboratory [32]. By contrast, Profile-2 TFs such as GATA1 and KLF1 known to be involved in β-globin activation and bind in the LCR [33], [34] were identified. Hierarchical clustering was used to visualize the genes identified by GSEA that are silenced and activated during fetal erythropoiesis (Figure 4B).

To provide evidence for a role of the novel TFs identified by GSEA in globin gene regulation, we performed *in silico* TESS and TFSEARCH analysis to locate predicted binding motifs in the β-locus (Figure S4 in File S1). Using the reference sequence file NG_000007.3 we investigated the LCR consisting of four erythroid-specific DNaseI hypersensitive sites 1 (HS1) to HS4. The LCR is known to bind TFs to mediate an enhancer function required for developmentally regulated globin gene expression [6]. In the LCR, we identified 15 TF binding motifs for the known globin regulator GATA1 and 5 motifs for NFE2 among others (Table 3 and Table S4); the novel TFs HES5 and HSF1 were also predicted to bind the LCR. A similar analysis for the HBG genes demonstrated binding motifs for Profile-1 genes such as GATA2 and novel factors TCFL7L2 and MXD3 not previously implicated in globin gene regulation (Table S5). Lastly, analysis for the HBB region identified binding motifs for 19 TFs such as the known regulator KLF1 and novel TF MAFB activated by day 56 (Table 3, Table S6). These data may provide insights into the role of novel DNA binding proteins in the γ/β-globin switch during fetal erythropoiesis.

**Table 3:** TESS/TFSEARCH predicted binding motifs across the β-globin locus.

Profile	GENE	Microarray Data^1^				Binding Motif^2^	Log-likelihood scores^3^	LCR^4^	γ-globin^4^	β-globin^4^
		D-21	D-42	D-49	D-56					
1	SP1	1.00	0.95	0.48	0.62	TGCAC	12	8	12	9
1	KLF4	1	0.97	1.01	0.78	CCYYTYYYTYNTTY	14	1	1	1
1	GATA2	1	0.95	0.57	0.32	AGATAA	12	2	2	2
1	CUX1	1	0.73	0.59	0.61	ATTGG	10	2	2	2
1	KLF11	1	0.95	0.57	0.32	TGGAATAT	12	1	1	-
1	HES5	1	0.95	0.57	0.32	CACGTG	12	1	1	-
1	FAF1	1	0.61	0.53	0.54	GGYMATTAA	16	-	1	-
1	TCF7L2	1	0.45	0.86	0.8	CTTTGAT	14	-	1	-
1	FHL2	1	0.84	0.72	0.45	AATGGGGA	12	-	1	-
1	MXD3	1	0.82	0.83	0.39	CATCTTGC	12	-	1	-
1	RBL1	1	0.4	0.46	0.37	GCGA	8	-	1	-
2	GATA1	1	0.81	1.32	1.4	RGAGATAA	16	15	21	10
2	ATF3	1	1.69	1.85	1.43	TGACGT	12	1	2	2
2	NFKB2	1	1.54	3.31	1.65	GGTAGTTCCC	20	1	-	1
2	BATF	1	1.44	1.65	1.53	CTCTGTGATGTCATGGTTT	17	-	-	1
2	KLF1	1	1.11	1.7	1.9	CCACACCCT	12	-	-	1
2	OLIG1	1	2.95	3.47	3.22	TCATATGG	12	-	-	1
2	MAFB	1	3.18	3.34	9.1	GCGGAAGT	10	-	-	1
2	OLIG2	1	20.81	22.44	24.91	TGTCCT	10	-	-	1
2	MXI1	1	3.91	5.65	4.99	CACGTG	12	-	-	9
2	CREB1	1	1.17	0.88	2.18	TGACG	10	5	2	6
2	NF-E2	1	1.26	1.68	1.36	TATA/GGGCAG	8	1	1	1
2	CREBBP	1	1.32	1.50	1.24	TTACGTAA	15	1	1	1
2	P300	1	1.58	2.37	1.55	GGGAGTG	14	2	1	1
2	Jun	1	2.72	1.85	2.52	TGACCCA	14	2	2	2
2	HSF1	1	1.35	9.20	2.07	AGAAC	7.8	2	2	2

1 Microarray data shown as fold change in expression from day 21 to day 56;

2 Nucleotide abbreviations: N = G, A or T; K = G or T, Y = T or C, M = A or C, R = A or G;

3 The Log-likelihood scores is a statistical measure representing the probability that a TF binding site exists in the region analyzed; we used a cutoff ≥7.0. The higher the score the more likely the predicted sequence binds the target TF indicated.

4 Number of binding sites identified in the different regions in the β-locus. For example there are 15, 21 and 10 predicted GATA1 binding sites in the LCR, γ-globin and β-globin regions respectively.

### Identification of TFNs involved in erythroid maturation

The next study was conducted to discover fetal erythroid TFNs using the genes defined by GSEA and predicted to bind the β-locus. Network analysis is a recently developed approach to study global gene regulatory pathways to define mechanisms of hemoglobin switching. The MiMI plugin for Cytoscape is a tool which integrates data from multiple well-known protein interaction databases including KEGG and Reactome into a network analysis as shown in Figure 4C (Figure S5 in File S1). We identified TFNs centered on GATA2 and KLF4 for Profile-1 genes and members of this network such as CREBBP and KLF6 regulated by KLF4. These data are consistent with a positive role of KLF4 in γ-globin regulation as previously published from our group [35]. Interestingly CREBBP is predicted to activate GATA2 and HDAC3. Perrine and colleagues demonstrated that knockdown of HDAC3 induces HbF expression due to displacement of this protein from the γ-globin promoter by short chain fatty acid derivatives [36]. How these TFNs controls globin gene regulation required additional studies.

The Cytoscape analysis, demonstrated that KLF1 and GATA1 serve as TFN hubs during late fetal erythropoiesis (Figure 4D and Figure S6 in File S1) consistent with their known role in hemoglobin switching. Downstream of KLF1, novel proteins such as CSNK2A1 which activates CREBBP but negatively regulates FLII were identified. Positive regulation of CSNK2A1 by KLF1 and the ability of CSNK2A1 to activate CREBBP by phosphorylation have been reported [31]. A replication study in β-thalassemia subjects show a correlation of single nucleotide polymorphisms in the CSNK2A1 gene correlate with fetal hemoglobin levels in this group [37].

### Different TFNs are involved in UCB versus adult erythropoiesis

**Figure 5:**
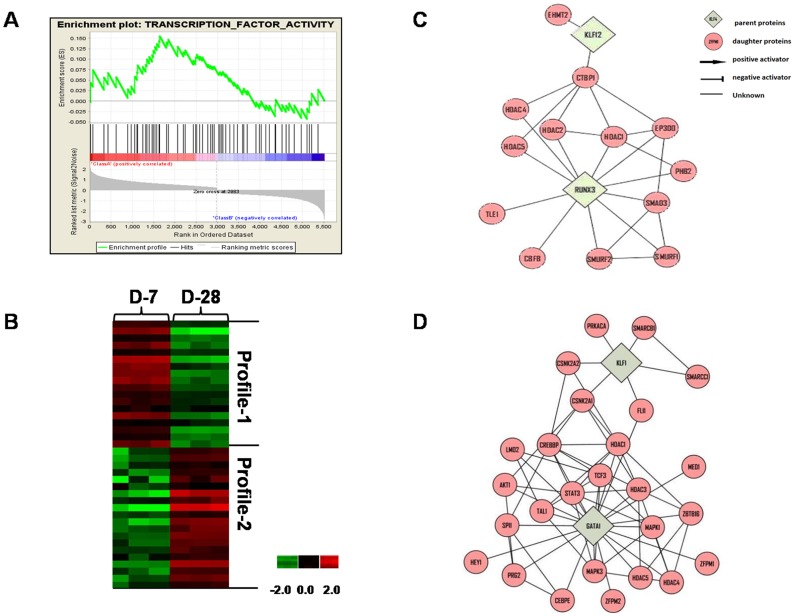
TF networks identified in erythroid progenitors generated from adult stem cells. A) Profile-1 (3142) and Profile-2 (5517) genes generated by PCA of data generated from adult stem cells. We compared day 7 to day 28 for GSEA analysis to produce ES and gene rank list as described in Figure 4A. We identified 18 Profile-2 (Class B) and 20 Profile-1 (Class A) TFs (Table S7). B) Hierarchical clustering analysis was performed for TFs identified by GSEA. The same color code was used as described in Figure 4B. C) Shown is a major TFN generated by Cytoscape analysis of Profile-1 genes. The key is included for interpretation of predicted regulatory interactions. D) Shown is a major TFN generated by Cytoscape analysis of Profile-2 genes. The interaction key is the same as in panel C.

To determine if unique mechanisms of globin gene regulation occur during fetal erythropoiesis, we compared TFNs defined using data generated from UCB versus adult bone marrow CD34^+^ cells [10] where the γ/β-globin switch occurred after day 42 and day 21 respectively (Figure 2A and Figure S2 in File S1). PCA using day 7 and day 28 data generated with adult erythroid progenitors produced 2649 Profile-1 and 2868 Profile-2 genes. Subsequent GSEA identified 14 Profile-1 (Class A) and 18 Profile-2 (Class B) TFs differentially expressed during adult erythropoiesis (Figure 5A; Table S7); hierarchical clustering demonstrated the genes silenced and activated during the culture period (Figure 5B).

Subsequent Cytoscape analysis defined TFNs centered on RUNX3 and KLF12 (Profile-1) before the γ/β-globin switch in adult progenitors (Figure 5C). KLF12 binds the CACCC boxes to regulate globin expression [38]. By contrast, RUNX3 interacts with Scl/Tal1 to control early stem cell development promoting commitment to the erythroid lineage and γ-globin activation [39]. Interestingly, the major Profile-2 TFNs generated for adult and fetal progenitors involve KLF1 and GATA1 (Figure 5D) however the downstream targets were less well defined in adult cells. These data support unique mechanisms of γ-globin regulation during erythropoiesis derived from fetal versus adult stem cells supported by different TFN hubs however the same factors KLF1 and GATA1 serve at TFN hubs after the switch.

### *In vivo* occupancy of TFs in the β-locus supported by ENCODE data

**Figure 6:**
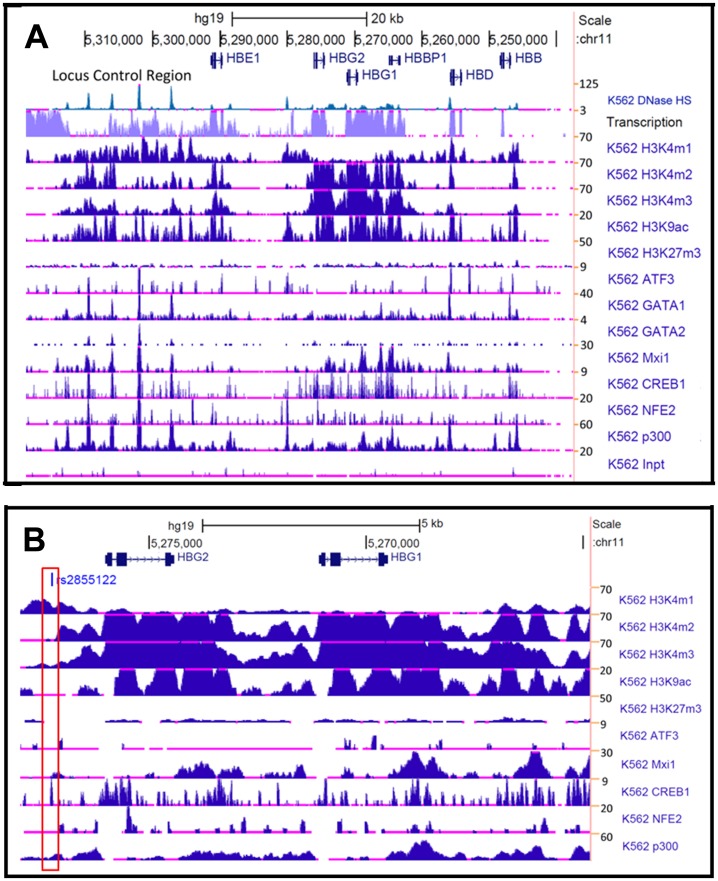
Data from the ENCODE project supports *in vivo* interaction of TFs. A) Shown are RNA-seq and ChIP-seq data for the β-locus (5,237,658 to 5,318,750) generated by the ENCODE consortium using K562 cells. Genes are indicated with arrows showing the direction of transcription. The numbers on the right side represents the maximum z-scores which correlate with signal strength. At the bottom of the panel, “K562 Inpt” refers to input chromatin incubated with negative control antibody. B) Shown are ChIP-seq and RNA-seq data for the HBG genes (5,264,860 to 5,277,966). The red box indicates the position for the cAMP responsive element at position -1222 relative to the HBG2 gene transcription start site.

For the TFs identified by GSEA and predicted to bind the β-locus by TESS and TFSEARCH analysis of fetal erythroblasts, we search for evidence of *in vivo* binding using data generated with K562 cells in the ENCODE database. Shown in Figure 6A is RNA-seq data demonstrating high transcriptional activity in the LCR and globin genes except *HBB* which is not expressed in K562 cells. ChIP-seq data related to histone modification, and occupancy of genomic regions by TFs was analyzed. The methylation status of histone H3 shows enhancer-associated marks (H3K4me1) at the LCR and 5′ of HBG2. Furthermore, acetylated histone H3 (H3K9ac) is present in conjunction with H3K4me1 and H3K4me2/3, whereas H3K27me3 (inactive chromatin) is detected at low levels supporting an active chromatin confirmation in the β-locus.

We next searched the ENCODE database for TFs predicted to bind in the β-locus in our analysis (Table 3). We observed ATF3 occupancy in the LCR and upstream of HBG2 which co-localized with the enhancer mark H3K4me1. Interestingly, MXI1 binding was detected in the LCR and HBG genes suggesting a role of MXI1 in regulating γ-globin expression; this DNA binding protein may be a novel regulator not previously identified. The ENCODE data also revealed a diffuse pattern of GATA1 binding throughout the β-locus with concentrated GATA2 binding in the LCR. NFE2 is another globin gene regulator [40] that showed high occupancy at the LCR and HBG genes. p300 which is associated with enhancer activity [41] showed high occupancy at the LCR and HBG genes co-localized with the H3K4me1/H3K9Ac active marks in the LCR. The ENCODE findings were recently expanded by Xu et al [42] demonstrating a major role of histone modifications in developmentally regulated globin gene expression. In erythroblasts derived from second trimester fetal liver cells, the highly expressed γ-globin gene was associated with activating histone marks H3K4me2/me3, H3K9ac and H3K27ac. By contrast, these marks are enriched around the adult δ- and β-globin genes in adult proerythroblast. These data support the combined role of lineage-specific regulators and co-regulator and stage-specific enhancers in developmentally regulated globin gene expression. Our data identified other potential co-regulators that function during erythropoiesis.

To gain a better perspective of the TFs implicated in HbF regulation, we studied factors at the genomic region covering the two HBG genes in detail (Figure 6B). Of note is the cAMP response element located at −1222 relative to the transcriptional start site of HBG2. This element is a positive regulatory sequence [43], [44] where transcription activity and H3K4me1 mark co-localize with CREB1 and p300 binding. Interestingly, ATF3 and MXI1 bind near the −1222 Gγ-globin cAMP response elements, supporting a function in globin gene regulation. Moreover, the presence of the H3K4me2/3 and H3K9Ac marks demonstrates open chromatin across the HBG genes that may delineate an active chromatin domain for γ-globin expression during fetal development. Xu et al [42] observed TF occupancy across the β-locus of proerythroblast derived from fetal liver and adult stem cells. They concluded that a set of developmental stage-specific enhancers that are marked by histone marks are functionally active in a stage-specific manner. Whether similar histone marks and/or novel TFs such as ATF3 and MXI1 play a role in hemoglobin switching during UCB-SC derived erythropoiesis requires further investigation.

### shRNA gene silencing identify novel repressors of HBG gene in primary erythroid cells

**Figure 7:**
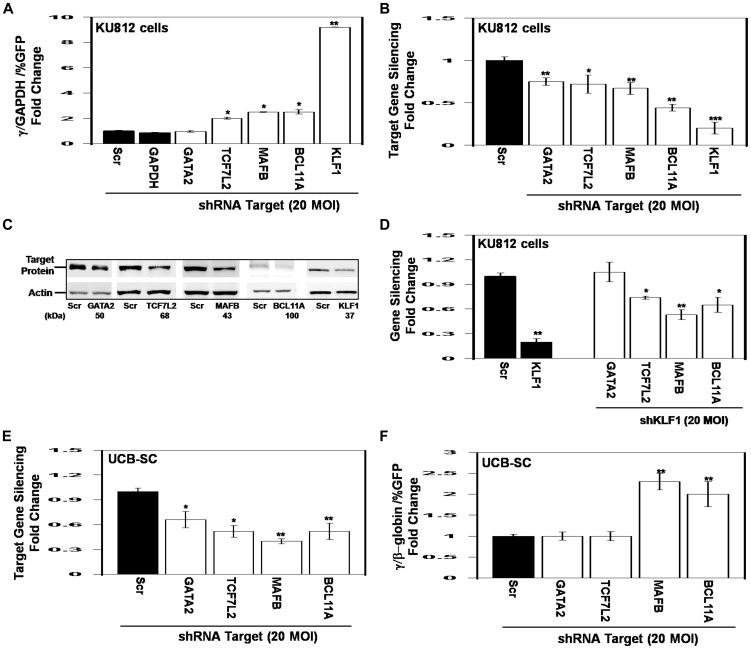
shRNA treatment of lead TFs augments γ-globin expression. KU812 cells were treated with shRNA lentiviral particles and selected with puromycin for 5 days followed by RT-qPCR and western blot analysis (see Materials and Methods). A) Shown is the fold change in γ-globin mRNA after treatment with the shRNA viral particles. γ-Globin expression was normalized by the percent of green fluorescent protein (GFP) positive cells representing transduction efficiency. B) RT-qPCR analysis was performed to confirm target gene silencing after shRNA treatment. ***p<0.0001 C) Western blot analysis was performed to confirm target gene silencing. D) RT-qPCR analysis of gene expression in shKLF1-treated KU812 cells to determine potential downstream gene targets. E) Erythroid progenitors generated from UCB-SC were transduced with shRNA lentiviral particles and selected with puromycin for 5 days followed by RT-qPCR and FACS analysis. Shown is the level of γ/γ+β-globin ratio after shRNA treatments. F) Target gene silencing was quantified after shRNA transductions.

To further support a functional role of TFs predicted to bind in the β-locus, we performed lentiviral-mediated shRNA gene silencing in KU812 cells [45] known to express the γ- and β-globin genes [46]. We focused our studies on TCF7L2 predicted to bind the HBG genes, MAFB predicted to bind HBB and GATA2 which binds across the β-locus, along with BCL11A and KLF1 known regulators of the γ/β-globin switch. shGATA2 transduction had no effect on γ-globin expression while knockdown of TCF7L2 and MAFB produced a 2.0-fold and 2.5-fold increase in γ-globin expression respectively (Figure 7A); as expected shBCL11A and shKLF1 treatment reactivated γ-globin expression 2.5-fold and 9.2-fold respectively corroborating a repressor effect of these factors. Target gene silencing by shRNA treatment was confirmed by RT-qPCR and western blot with 50–80% reduction in target gene expression (Figure 7B, 7C). Since KLF1 has been demonstrated to be a potent repressor of γ-globin expression, we also performed qPCR using RNA isolated from shKLF1 treated KU812 cells. KLF1 knockdown reduced BCL11A expression along with TCF7L2 and MAFB (Figure 7D) suggesting they might also be downstream targets. The regulation of BCL11A by KLF1 is consistent with previously published data [20], [21]. By contrast, there was no significant change in GATA2 expression suggesting this factor is not regulated by KLF1.

Lastly, we conducted shRNA studies in primary erythroid cells generated from UCB-SC. On day 49 we treated erythroid progenitors with shTCF7L2, shMAFB, shGATA2, shBCL11A and scrambled controls. We chose this time point because γ-globin expression is low and there remain 40% polychromatophilic erythroblasts (Figure 1C) capable of responding to shRNA treatment. Puromycin was added on day 51 for 5 days and then cells were harvested for RT-qPCR and FACS analyses. We observed 35–70% target gene silencing (Figure 7E) which produced an increase in the γ/γ+β-globin ratio by 2.3-fold and 2-fold by shMAFB and shBCL11A treatment respectively (Figure 7F). The level of β-globin gene expression was not altered significantly by shRNA treatment (data not shown). MAFB is a leucine zipper TF predicted to bind the HBB region which plays a pivotal role in regulating lineage-specific hematopoiesis by repressing transcription of erythroid specific genes in myeloid cells [47] by interaction with the binding partner c-Ets1. Interestingly, the shRNA data suggest MAFB is a novel repressor of γ-globin however its molecular mechanism remains to be defined.

The findings suggest our bioinformatics analysis identified lead TFs not previously implicated in globin gene regulation however additional experimental data are required to confirm their *in vivo* functional role. In recent years, stem cells isolated from UCB have been investigated because of their high proliferation capacity and lack of tumorogenicity. The goal of our study was to delineate the transcriptome and unique TFNs involved in fetal erythropoiesis. We observed a higher proliferative capacity of UCB-CD34^+^ stem cells and normal erythroid maturation. In fetal erythroid progenitors the γ/β-globin gene switch occurred after day 42 demonstrating prolonged γ-globin gene expression compared to adult erythroid progenitors grown in the same culture conditions. Microarray analysis followed by GSEA and Cytoscape analysis defined major TFNs around KLF4 and GATA2 before γ-globin silencing and KLF1 and GATA1 after β-globin activation. shRNA-mediated gene silencing in erythroid progenitors derived from UCB-SC implicated MAFB as novel repressors of γ-globin expression consistent with our model that Profile-2 TFs are negative regulators. We also identified other novel TFs such as HES5, ATF3, MXD3 and CUX1 among others (Table 3) predicted to bind the γ-globin promoter that can be analyzed by shRNA studies in fetal erythroblasts to define a functional role in globin gene regulation.

Fetal hemoglobin is a potent inhibitor of sickle hemoglobin polymerization and compensate for the globin chain imbalance in β-thalassemia. Clinical evidence indicates a modest increase in HbF, is a major modifier of the clinical phenotype and mortality in sickle cell disease [48]. Although hydroxyurea induces HbF in adults [12] and children [13], pharmacologic agents that specifically target hemoglobin switching have not been developed. Thus our results and others support developmental-stage specific control of globin gene expression that has important implications for the development of gene-based therapies for sickle cell disease and β-thalassemia. Several criteria must be met to define molecular targets of HbF induction including direct γ-globin silencing during erythropoiesis with limited off target effects and normal progression of hematopoiesis. For example, c-Myb may be a prospective target but its role in maintenance and differentiation of hematopoietic stem cells [49] raise concerns whether a safe therapeutic window can be achieved. Similar concerns about the development of BCL11A and KLF1 as therapeutic target exist. Recent studies to define the molecular mechanism of γ-globin regulation by BCL11A suggest targeting an erythroid specific enhancer in the first intron make this factor a promising target [50]. However there remains a need to identify other TFs that directly target γ-globin promoter regulatory elements and are expressed in a stage-specific manner to expand the repertoire of DNA-binding proteins that mediate HbF induction. The study herein and others [51] work towards this goal.

## Supporting Information

Table S1 **Microarray normalized raw data for day 21, 42, 49 and 56.**
(XLSX)

Table S2 **Profile 1 and Profile-2 principal component analysis data.**
(XLSX)

Table S3 **Micorarray and RT-qPCR confirmation.**
(XLSX)

Table S4 **TESS/TFSEARCH analysis for locus control region.**
(XLSX)

Table S5 **TESS/TFSEARCH analysis for HBG region.**
(XLSX)

Table S6 **TESS/TFSEARCH results for HBB globin gene.**
(XLSX)

Table S7 **Adult stem cell Profile-1 (Class A) and Profile-2 (Class B) transcription factors.**
(XLSX)

File S1 **Supplementary figures.**
(PPT)
