# Supplementary material for: Characterization of Transcription Factor Networks Involved in Umbilical Cord Blood CD34+ Stem Cells-Derived Erythropoiesis
Source: PLoS One. 2014 Sep 11;9(9):e107133. doi: 10.1371/journal.pone.0107133 (PMC4161396; doi:10.1371/journal.pone.0107133)
Supplement: File S1 — Supplementary figures. (PPT) [file pone.0107133.s008.ppt]

## Slide 1
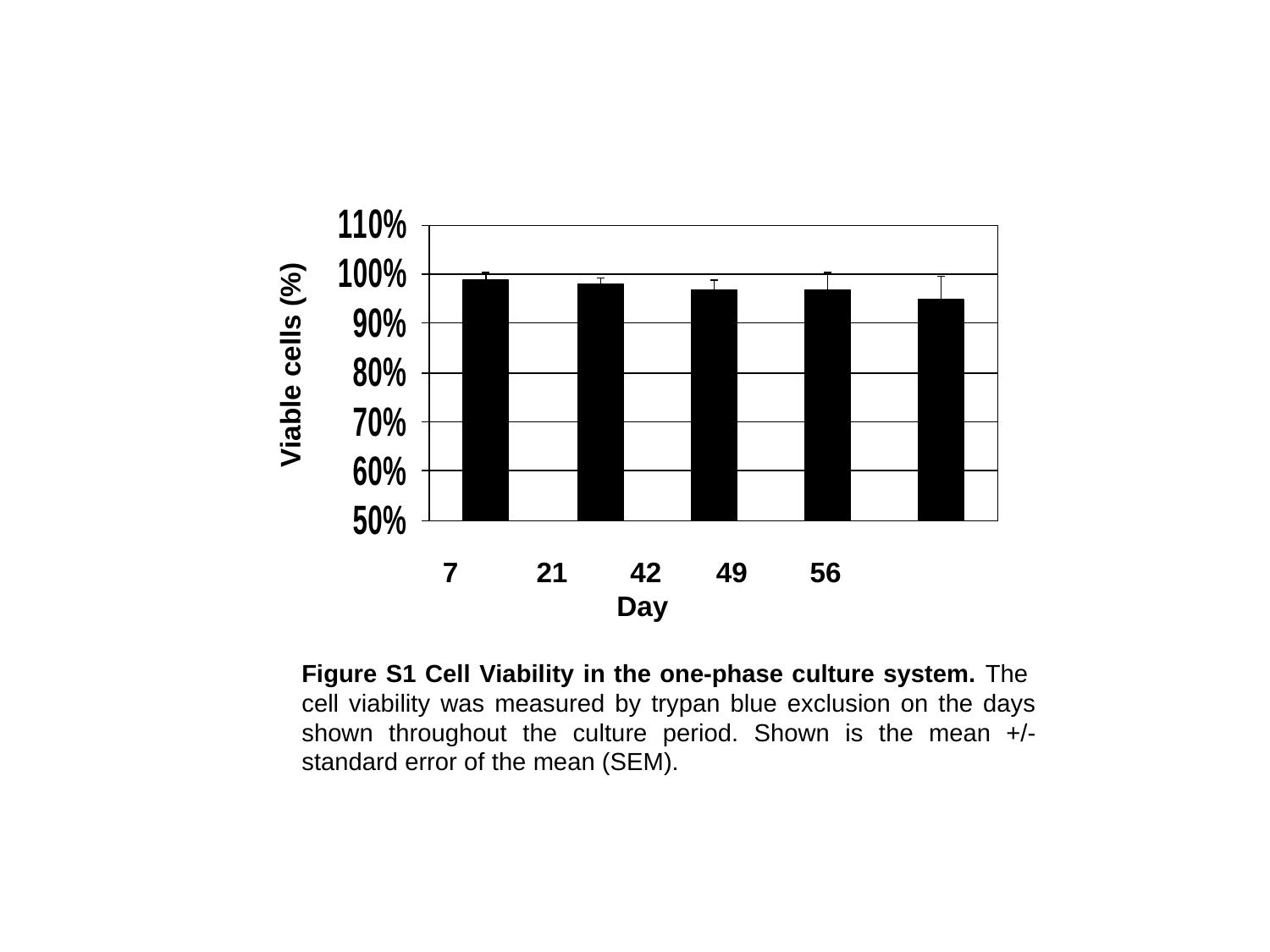

Viable cells (%)
7 21 42 49 56
	 Day
Figure S1 Cell Viability in the one-phase culture system. The cell viability was measured by trypan blue exclusion on the days shown throughout the culture period. Shown is the mean +/- standard error of the mean (SEM).

## Slide 2
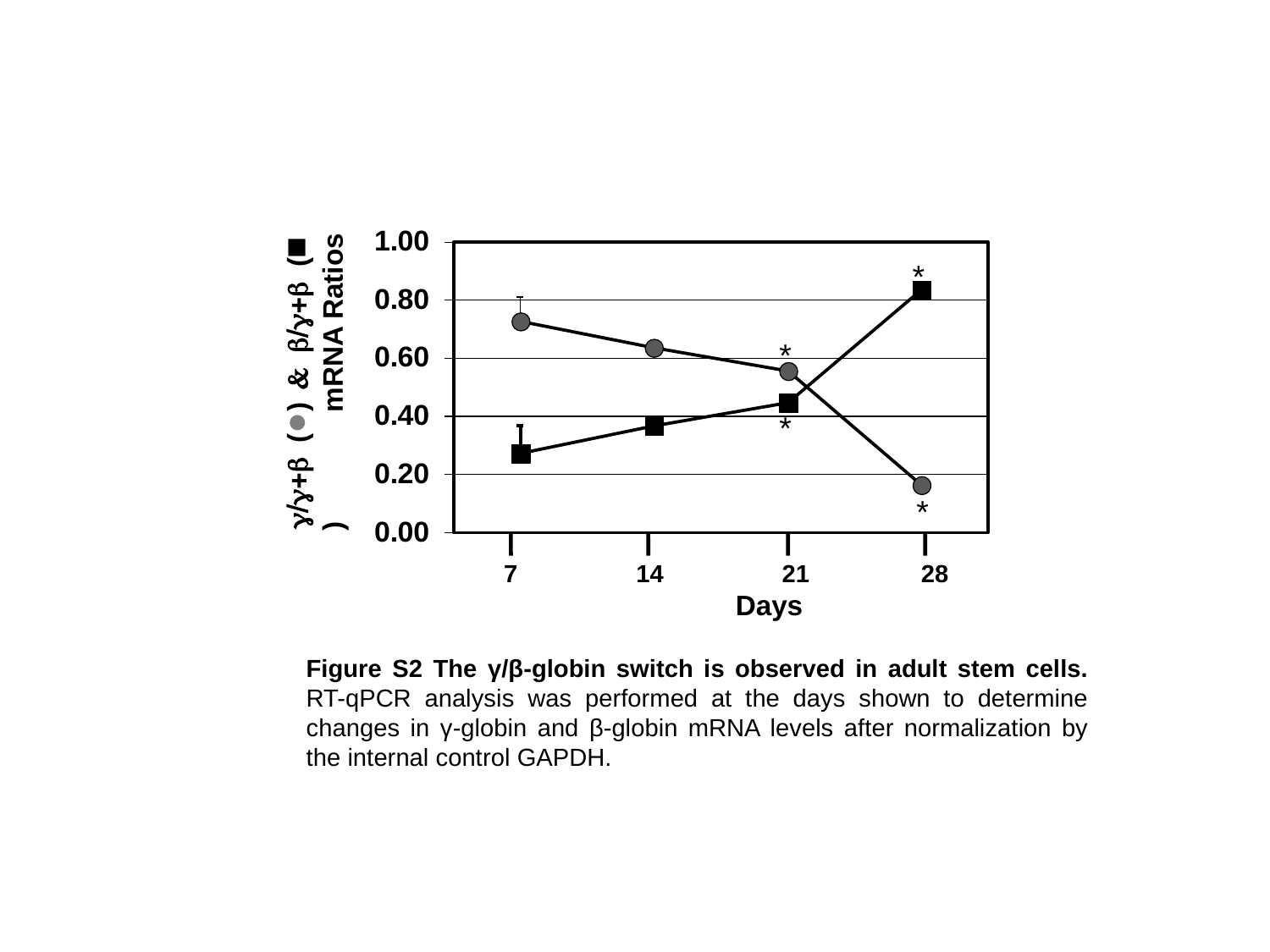

*
 (●)   (■) mRNA Ratios
*
*
*
7 14 21 28 		 Days
Figure S2 The γ/β-globin switch is observed in adult stem cells. RT-qPCR analysis was performed at the days shown to determine changes in γ-globin and β-globin mRNA levels after normalization by the internal control GAPDH.

## Slide 3
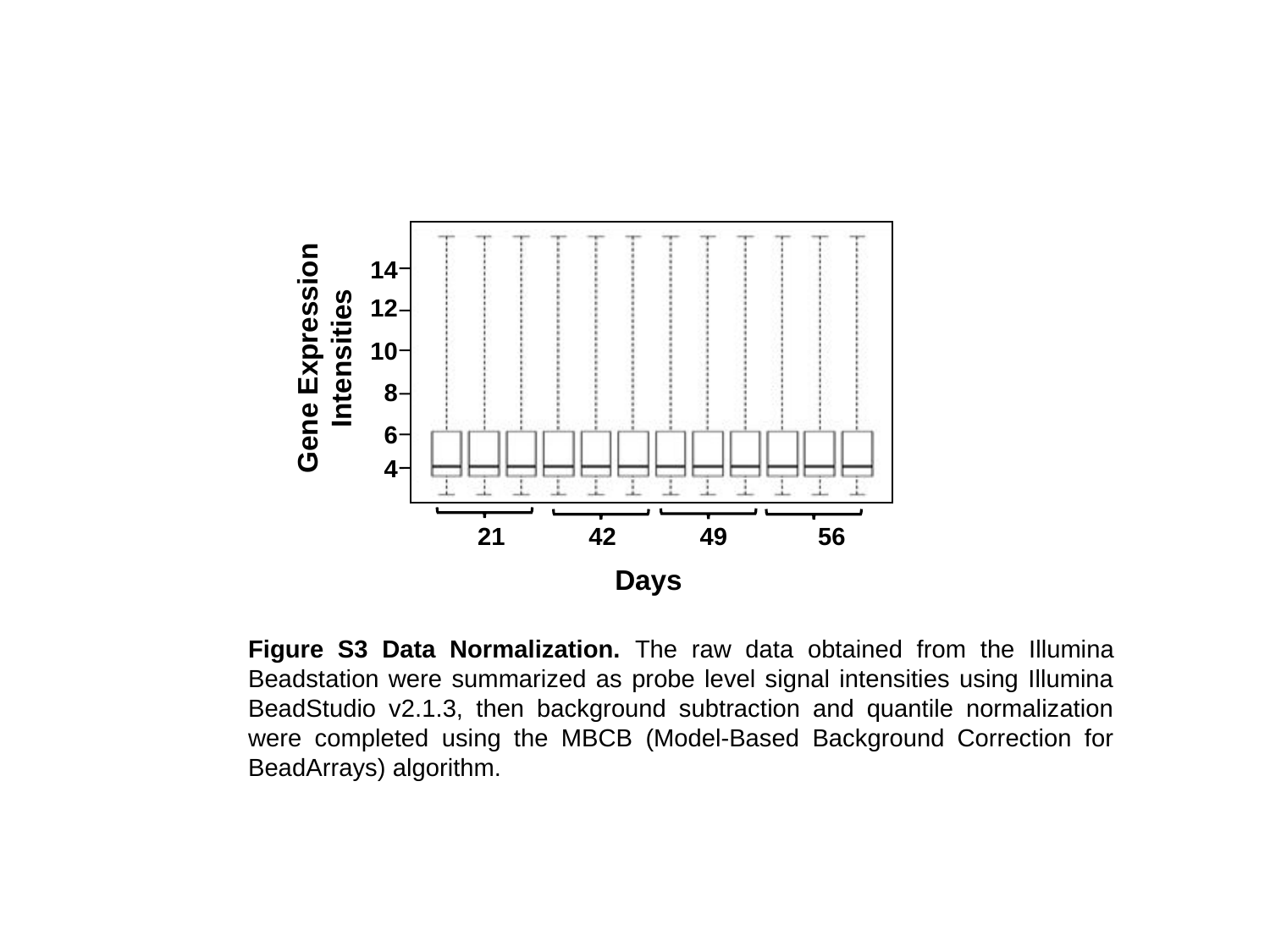

14
12
10
8
6
4
Gene Expression Intensities
 21 42 49 56
Days
Figure S3 Data Normalization. The raw data obtained from the Illumina Beadstation were summarized as probe level signal intensities using Illumina BeadStudio v2.1.3, then background subtraction and quantile normalization were completed using the MBCB (Model-Based Background Correction for BeadArrays) algorithm.

## Slide 4
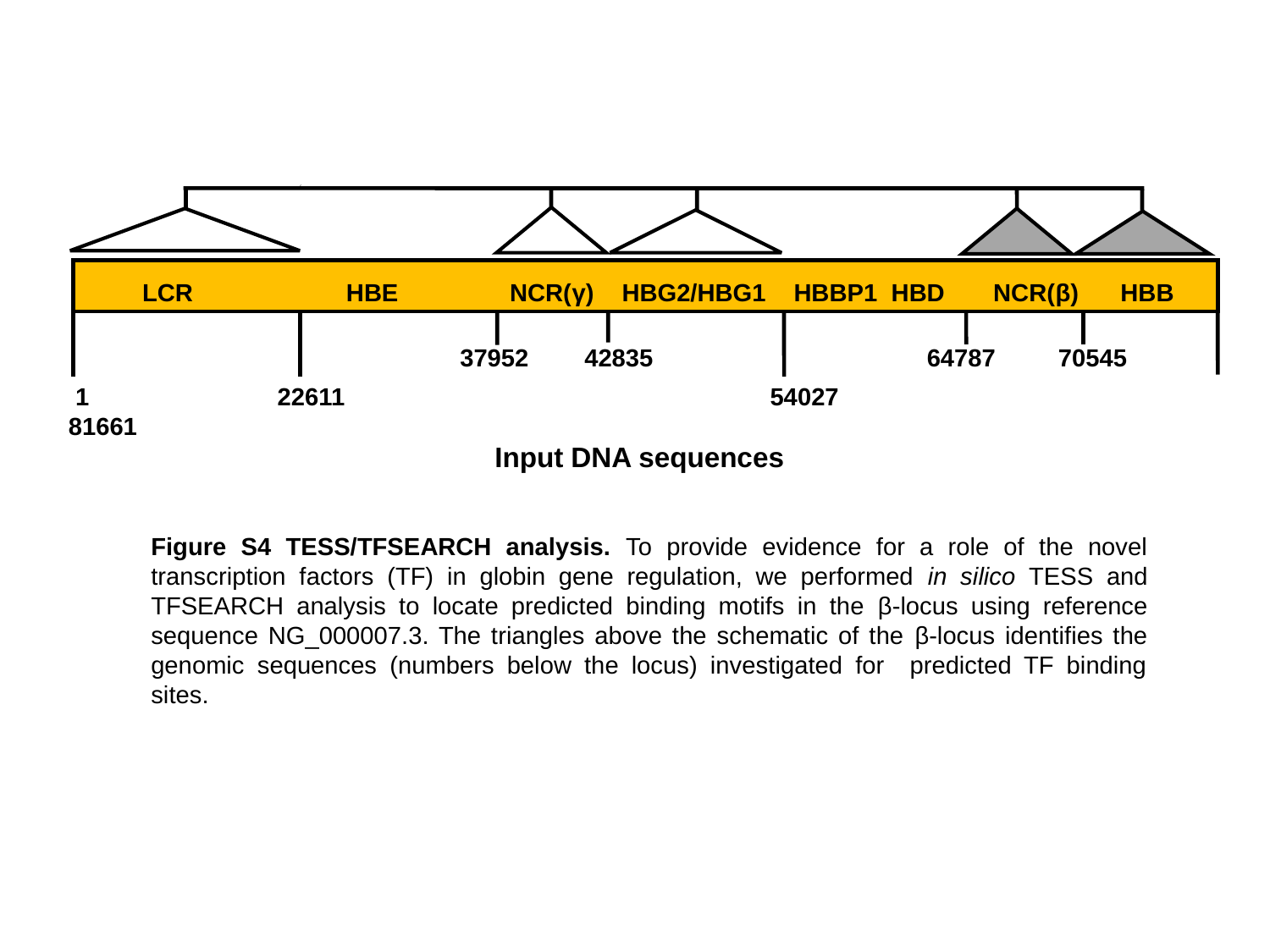

LCR HBE NCR(γ) HBG2/HBG1 HBBP1 HBD NCR(β) HBB
 37952 42835
 64787 70545
 1 22611 54027 81661
 Input DNA sequences
Figure S4 TESS/TFSEARCH analysis. To provide evidence for a role of the novel transcription factors (TF) in globin gene regulation, we performed in silico TESS and TFSEARCH analysis to locate predicted binding motifs in the β-locus using reference sequence NG_000007.3. The triangles above the schematic of the β-locus identifies the genomic sequences (numbers below the locus) investigated for predicted TF binding sites.

## Slide 5
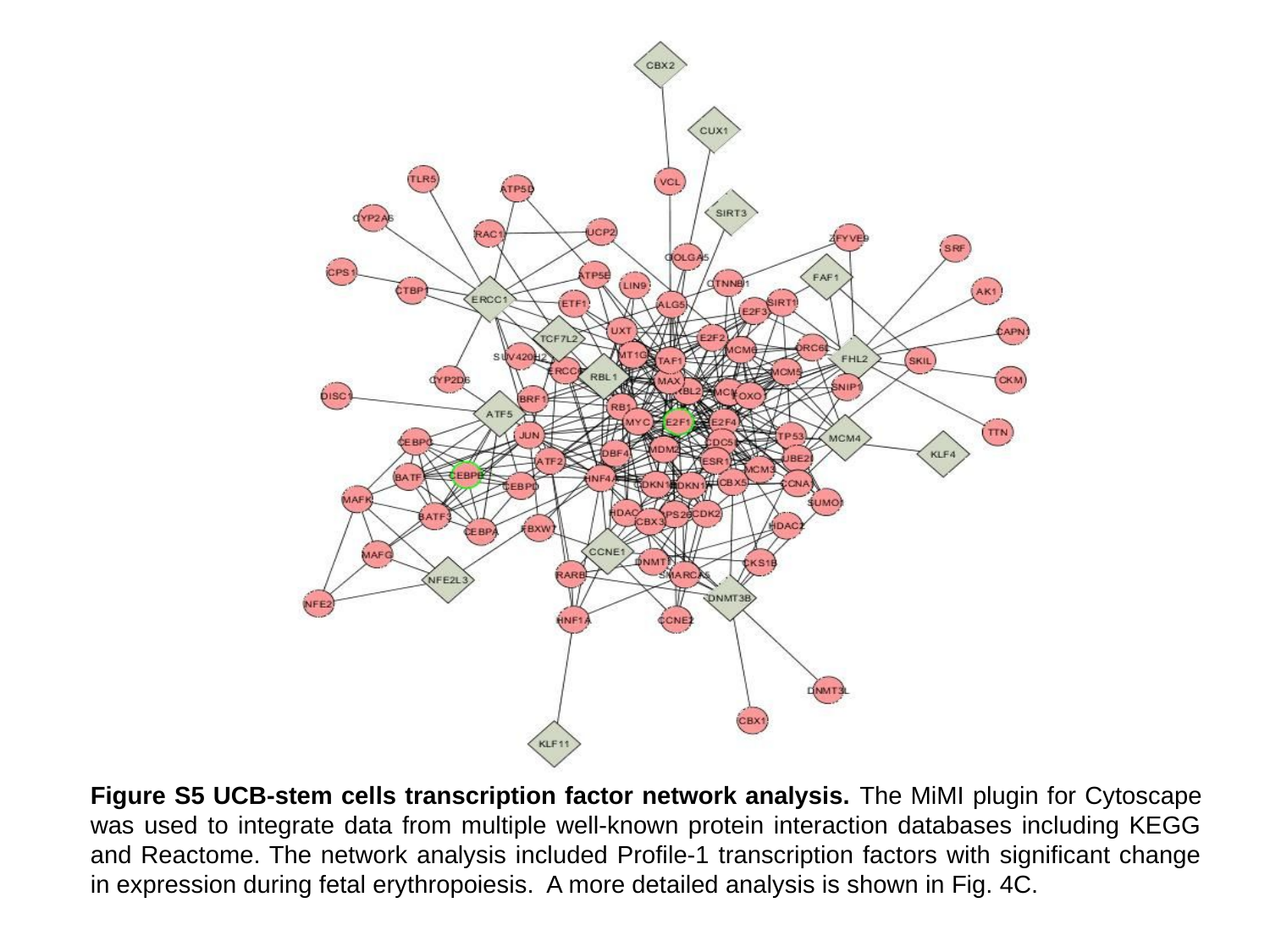

Figure S5 UCB-stem cells transcription factor network analysis. The MiMI plugin for Cytoscape was used to integrate data from multiple well-known protein interaction databases including KEGG and Reactome. The network analysis included Profile-1 transcription factors with significant change in expression during fetal erythropoiesis. A more detailed analysis is shown in Fig. 4C.

## Slide 6
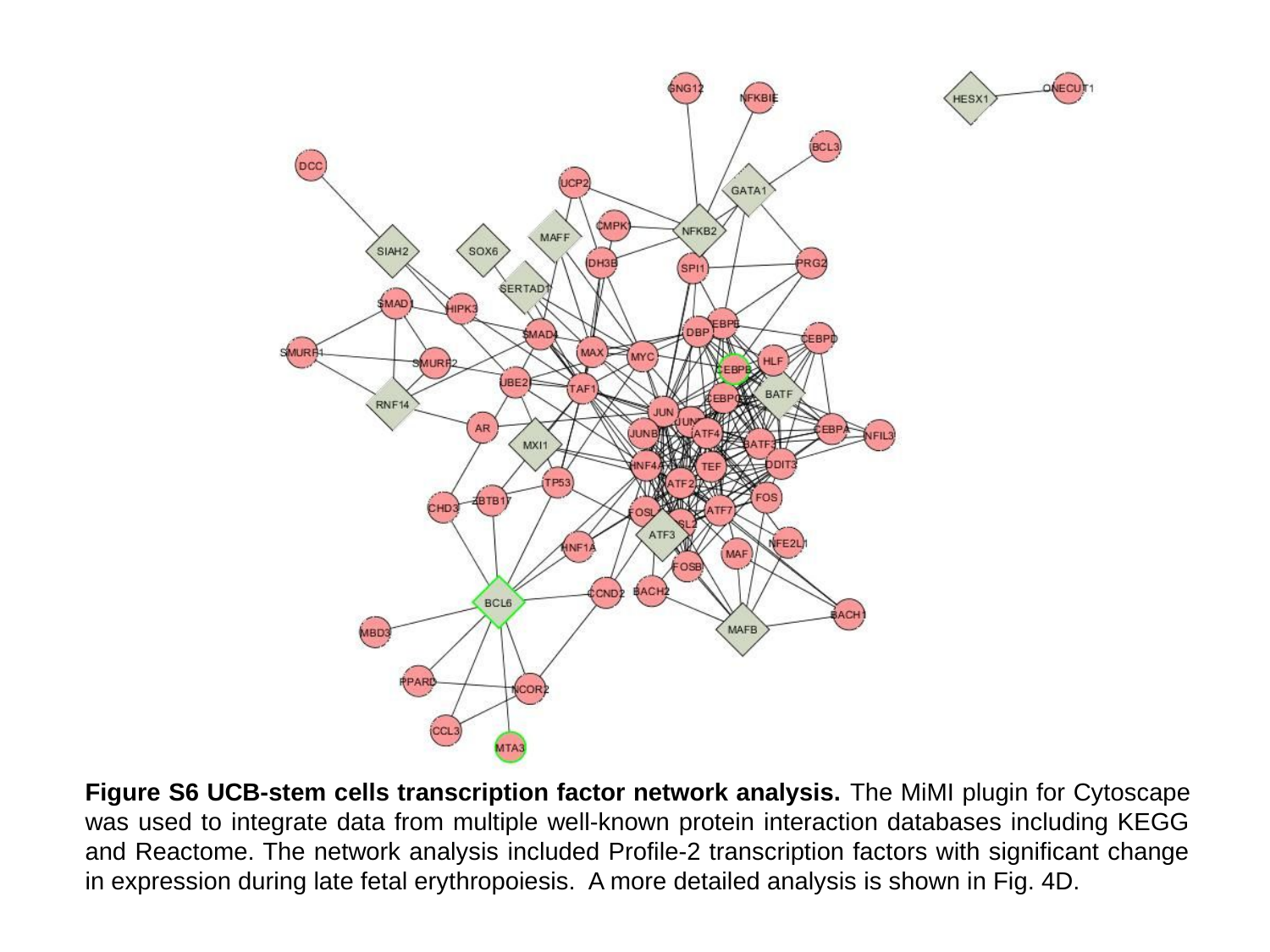

Figure S6 UCB-stem cells transcription factor network analysis. The MiMI plugin for Cytoscape was used to integrate data from multiple well-known protein interaction databases including KEGG and Reactome. The network analysis included Profile-2 transcription factors with significant change in expression during late fetal erythropoiesis. A more detailed analysis is shown in Fig. 4D.
